# Loss of RAB1B promotes triple-negative breast cancer metastasis by activating TGF-β/SMAD signaling

**DOI:** 10.18632/oncotarget.3877

**Published:** 2015-04-19

**Authors:** Hong-Lin Jiang, He-Fen Sun, Shui-Ping Gao, Liang-Dong Li, Xin Hu, Jiong Wu, Wei Jin

**Affiliations:** ^1^ Department of Breast Surgery, Key Laboratory of Breast Cancer in Shanghai, Collaborative Innovation Center of Cancer Medicine, Fudan University Shanghai Cancer Center, Shanghai, China; ^2^ Department of Oncology, Shanghai Medical College, Fudan University, Shanghai, China

**Keywords:** RAB1B, triple-negative, metastatic breast cancer, TGF-β/SMAD signaling

## Abstract

Triple-negative breast cancer (TNBC) is a highly aggressive tumor subtype associated with a poor prognosis. The mechanism involved in TNBC progression remains largely unknown. To date, there are no effective therapeutic targets for this tumor subtype. In this study, by performing quantitative proteomic analyses in highly metastatic and parental breast cancer cell line, we found that RAB1B, a member of the RAS oncogene family, was significantly down-regulated in highly metastatic breast cancer cells. Moreover, down-regulation of RAB1B was also found to promote the proliferation and migration of TNBC cells *in vitro* and *in vivo*. Mechanistically, loss of RAB1B resulted in elevated expression of TGF-β receptor 1 (TβR1) through decreased degradation of ubiquitin, increased levels of phosphorylated SMAD3 and TGF-β-induced epithelial-mesenchymal transition (EMT). Furthermore, low RAB1B expression correlated with poor prognosis in breast cancer patients. Taken together, our findings reveal that RAB1B acts as a metastasis suppressor in TNBC by regulating the TGF-β/SMAD signaling pathway and RAB1B may serve as a novel biomarker of prognosis and the response to anti-tumor therapeutics for patients with TNBC.

## INTRODUCTION

Triple-negative breast cancer (TNBC) is an invasive type of breast carcinoma that lacks expression of the estrogen receptor (ER) and progesterone receptor (PR) as well as human epidermal growth factor receptor 2 (HER2) amplification. TNBCs constitute approximately 10-17% of all invasive breast carcinomas and tend to more frequently affect younger patients [1-6]. TNBC tumors are also generally larger in size, of a higher grade, present lymph node involvement at the time of diagnosis, and are biologically more aggressive [1]. Due to the heterogeneity of this disease and the absence of well-defined molecular targets [7-9], the treatment of TNBC has remained challenging. Indeed, less than 30% of women with metastatic TNBC survive for 5 years despite normative adjuvant chemotherapy [4]. Clearly, there is an urgent need for useful biomarkers that can predict the metastatic potential of TNBC and serve as prognostic indicators or targets for treatment.

In our previous study, The MDA-MB-231HM was developed from the parental MDA-MB-231 cell line via the tail vein in mice for four cycles and we have patent application for the cell line (the patent number: 200910174455.4). Accordingly, MDA-MB-231HM cells exhibit increased invasiveness compared to parental MDA-MB-231 cells (Supplementary Figure S1). Because of the similar genetic background of these cells, they provide a unique model for identifying candidate metastasis-associated biomarkers and potential therapeutic targets for TNBC. In the current study, to comprehensively understand the roles of metastasis-related proteins in TNBC progression, iTRAQ labeling technology followed by nanoscale high-performance liquid chromatography-tandem mass spectrometry (nano-HPLC-MS/MS) was used to compare the whole-cell proteome profile of the two cell lines [10]. Our analysis identified RAB1B as a differentially expressed protein that was associated with the metastatic potential of TNBC.

RAB GTPase proteins constitute one of the largest subfamilies of small GTPases, which play a master role in regulating intercellular vesicle trafficking in endocytic pathways. Dysregulation of RAB gene expression is involved in some types of human tumors, and previous studies have reported that RAB25 overexpression drives ovarian cancer progression [11] and that up-regulation of RAB5A and RAB7 occurs in thyroid-associated adenomas [12], suggesting a role for RAB proteins in the progression of tumors. RAB1B is known for its role in vesicle transport from the endoplasmic reticulum (ER) to the Golgi complex; however, few reports have studied RAB1B in the context of human tumors [13]. In this work, we show, for the first time, that RAB1B is a suppressor of triple-negative breast cancer metastasis.

## RESULTS

### RAB1B expression is down-regulated in highly metastatic breast cancer cells

**Figure 1:**
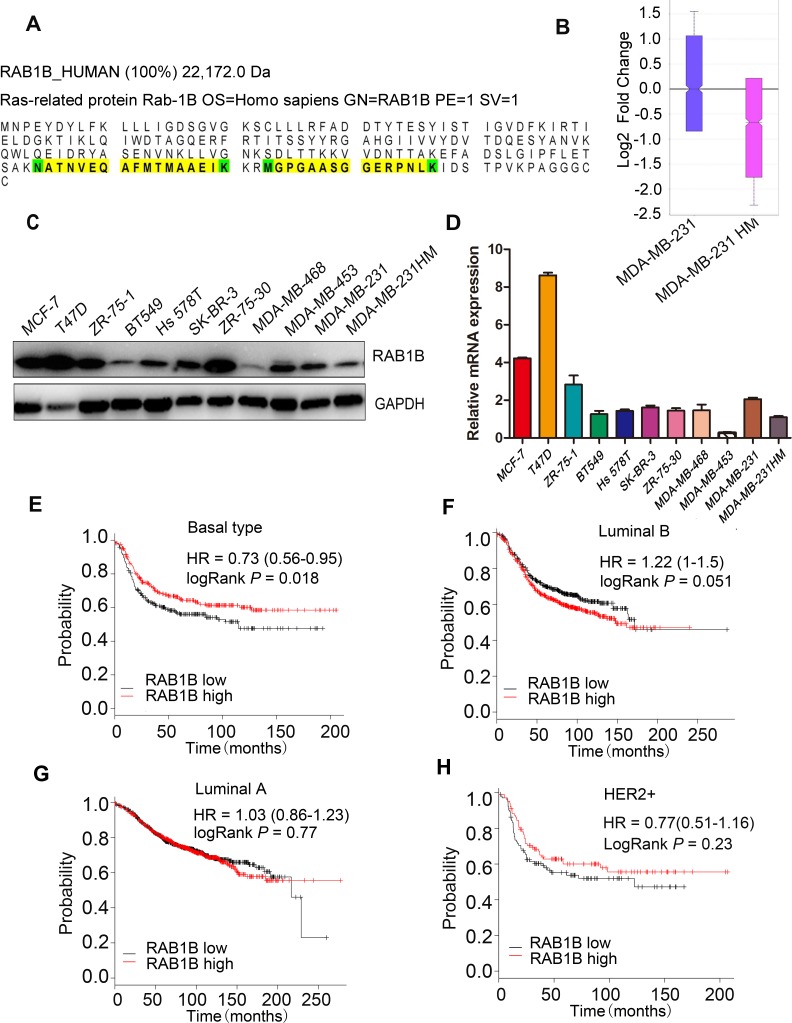
RAB1B is down-regulated in highly metastatic breast cancer cells **A.** The peptide sequence of RAB1B detected using iTRAQ labeling. **B.** The fold change ratio of the breast cancer cell lines MDA-MB-231 and MDA-MB-231HM by iTRAQ labeling based on quantitative proteomic analyses. **C.** and **D.** The protein and mRNA expression of RAB1B in breast cancer cell lines. **E.**, **F.**, **G.** and **H.** Survival analysis of RAB1B expression in patients with basal subtype, luminal B, luminal A and HER-2 positive breast cancer, performed using the online Kaplan-Meier plotter. (HER-2: human epidermal growth factor receptor 2).

Using the iTRAQ labeling method in our model system, we found that RAB1B was significantly down-regulated in MDA-MB-231HM compared with MDA-MB-231 cells (Figure 1A, 1B). In addition to these two breast cell lines, a panel of breast cell lines was also evaluated to observe the general trend of gene expression. In accordance with the results observed in our iTRAQ experiments, RAB1B expression was generally observed in weakly metastatic cell lines (MCF-7, T47D, ZR-75-1, ZR-75-30, BT-549, SK-BR-3), but was down-regulated in highly metastatic cell lines (Hs 578T, MDA-MB-231, MDA-MB-231HM) (Figure 1C, 1D). We also studied the prognostic value of RAB1B (Affy ID: 220964_s_at) using the online Kaplan-Meier plotter for survival analysis. For the patients with basal subtype tumors, low RAB1B expression had a negative impact on relapse-free survival (Figure 1E). However, RAB1B expression did not correlate with relapse-free survival in patients with luminal A, Luminal B and Her-2 positive breast tumors (Figure 1F-1H). Taken together, these data suggest that RAB1B may represent a novel biomarker worthy of further investigation.

### Loss of RAB1B expression in breast cancer cells promotes cell proliferation and cell migration *in vitro*

**Figure 2:**
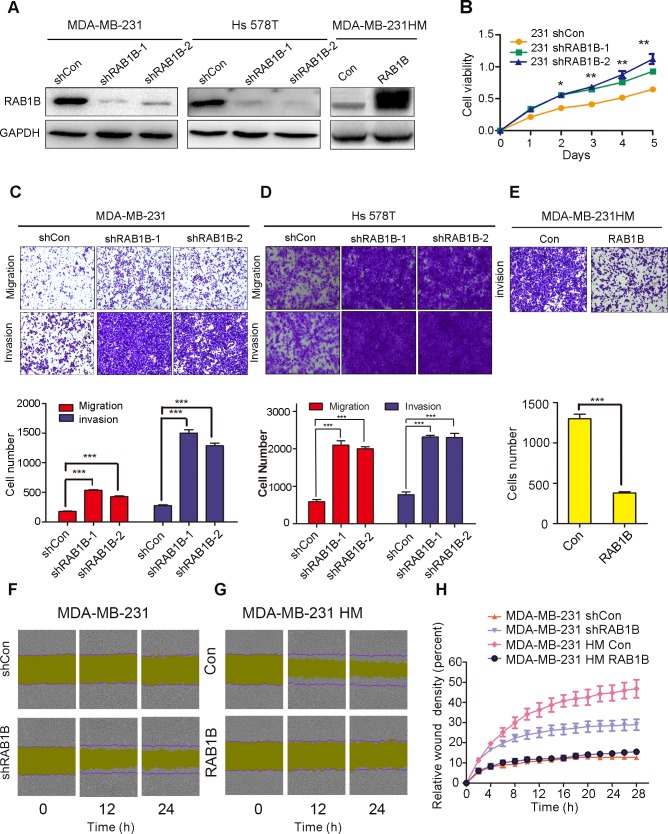
Low RAB1B expression promotes breast cancer metastasis *in vitro* **A.** RAB1B was stably knocked down in parental MDA-MB-231 cells and Hs 578T cells using two independent shRNA constructs and was constitutively overexpressed in highly metastatic MDA-MB-231HM cells. **B.** Proliferation assays (CCK-8) in MDA-MB-231 cells with knocked down RAB1B expression, *P < 0.05, **P < 0.01. **C.**, **D.** and **E.** The migration and invasion ability of each cell line was evaluated by transwell assay *in vitro*. The left panels show photos of representative fields (100× magnification) of invasive cells, and the right panel shows histograms of the results. The statistical analysis was performed using Student's *t*-test (*n* = 3). The error bars represent the SD, ***P < 0.001. **F.** and **G.** Effects of RAB1B on migration of MDA-MB-231 and MDA-MB-231HM cells in wound-healing assay. The purple lines indicate the initial scratch wound location and the yellow area showed the scratch wound mask. Images were captured at the indicated times after wounding. **H.** The effect of RAB1B on the percent of relative wound density in the indicated time point. Error bars indicate SEM.

To further investigate whether RAB1B is involved in human TNBC metastasis, we used two independent shRNAs targeting RAB1B in MDA-MB-231 and Hs 578T cells. An empty vector (shCon) served as the control. Western blot analysis demonstrated that both shRNA constructs strongly reduced the level of RAB1B protein (Figure 2A). Furthermore, we generated a RAB1B stably overexpressing MDA-MB-231HM cell line (Figure 2A). Next, we evaluated the effect of down-regulated RAB1B on the malignant phenotype of breast cancer cells *in vitro*. These results showed that RAB1B knockdown significantly promoted cell proliferation in MDA-MB-231 cells(Figure 2B). Additionally, a significant increase in migration and invasion ability was observed in RAB1B knockdown cells (Figure 2C, 2D). Using a reverse-complimentary approach, we demonstrated that elevated RAB1B expression suppressed the invasion ability of cells in a transwell assay (Figure 2E). Moreover, depleting RAB1B activated MDA-MB-231 cells to migrate in a wound-healing assay, whereas restoration of RAB1B in MDA-MB-231HM cells decreased cell migration ability (Figure 2F-2H). These results suggest a potential role for RAB1B in suppressing cell proliferation and metastasis *in vitro*.

### Down-regulation of RAB1B activates TGF-β signaling by elevating TGF-β receptor 1 (TβR1) protein levels

**Figure 3:**
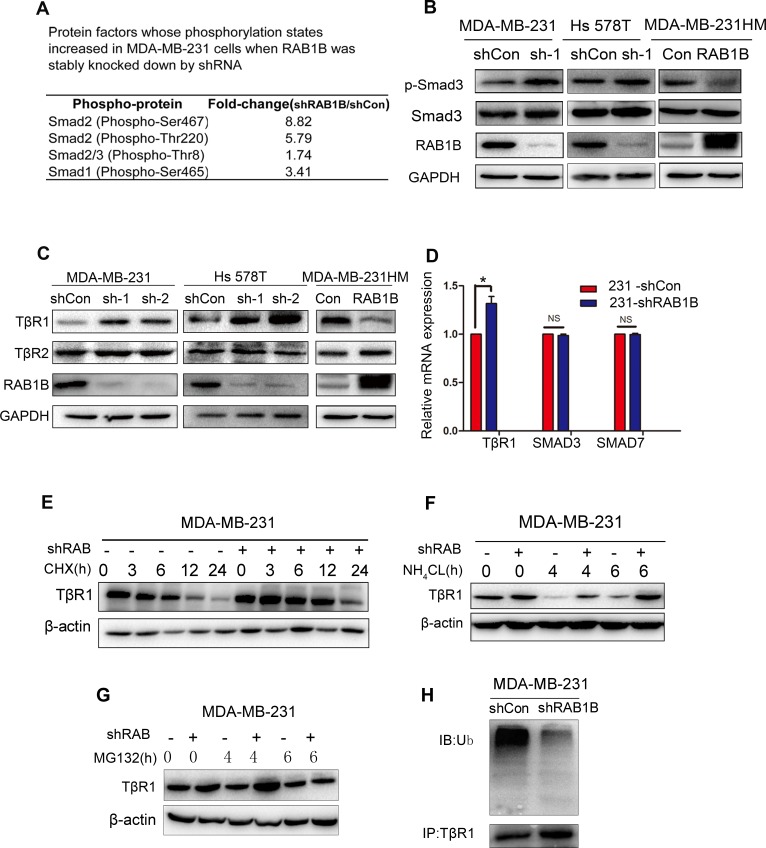
Loss of RAB1B activates TGF-β/SMAD signaling by suppressing TβR1 degradation **A.** Selected protein factors whose phosphorylation states were increased in MDA-MB-231 shCon and shRAB1B cells. **B.** Immunoblot analysis of p-Smad3 and Smad3 expression in RAB1B knockdown and overexpressing cells. **C.** Western blot analysis of TβR1 and TβR2 expression in RAB1B knockdown and overexpressing cells. **D.** Real-time PCR analysis of TβR1, SMAD3 and SMAD7 mRNA levels in MDA-MB-231 shCon and shRAB1B cells. Data are presented as the mean ± SD. (*n* = 3), *P < 0.05. **E.**, **F.** and **G.** Western blot analysis of TβR1 expression in MDA-MB-231 shCon and shRAB1B cells after incubation with 50 μM CHX **E.**, 10 μM NH_4_CL **F.** and 10 μM MG132 **G.** for the indicated number of hours. **H.** MDA-MB-231 shCon and shRAB1B cells were treated with 10 μM MG132 for 4 h. Following cell harvest, proteins were immunoprecipitated with an anti-TβR1 antibody and detected using a polyubiquitin antibody. (Ub: ubiquitin).

To explore the molecular mechanism underlying enhanced metastasis *in vitro* due to decreased RAB1B expression, we surveyed the potential signaling pathways using a phospho-antibody microarray assay. Analysis of the array revealed the induction in protein expression of several key components of the TGF-β pathway as a result of depletion of RAB1B, including SMAD2 (phospho-Ser467), SMAD2 (phospho-Thr220) and SMAD1 (phospho-Ser465), which were increased by 8.82-fold, 5.79-fold and 3.41-fold, respectively (Figure 3A). We next investigated the effect of RAB1B on key components of the TGF-β pathway, and we found that depleting RAB1B resulted in a strong induction of TβR1 protein levels (Figure 3C). As a result of TβR1 up-regulation, although SMAD2 (phospho-Ser467) expression was not significantly up-regulated as observed in the microarray results (data not shown), SMAD3, another key mediator of TGF-β signaling, showed a significant increase in phosphorylation (phospho S423+S425). In contrast, RAB1B overexpression in MDA-MB-231HM cells markedly down-regulated the protein level of TβR1 and p-SMAD3 (Figure 3B and 3C). Moreover, we measured the mRNA expression of other components of the TGF-β pathway, such as SMAD3 and SMAD7, although no positive results were obtained (Figure 3D). These findings indicate that down-regulation of RAB1B activates TGF-β signaling by elevating TβR1 protein levels.

### RAB1B correlates with TβR1 degradation

We observed a significant increase in TβR1 protein expression following RAB1B knockdown. However, there was only a modest up-regulation of TβR1 mRNA expression upon RAB1B knockdown (Figure 3D). These results suggest that RAB1B predominantly suppresses TβR1 in a post-transcriptional manner. To confirm whether RAB1B is associated with the TβR1 protein degradation pathway, MDA-MB-231 cells were incubated with cycloheximide (CHX). Compared with RAB1B knockdown cells (MDA-MB-231 shRAB1B), TβR1 was degraded more rapidly and became less detectable within 6 h of CHX treatment in the control cell line (MDA-MB-231 shCon) (Figure 3E). Furthermore, treatment of these cells with the proteosomal inhibitor MG132 increased the stable TβR1 protein level, suggesting that TβR1 is degraded through the ubiquitin-proteosome system (UPS) (Figure 3G). Indeed, in RAB1B stably depleted MDA-MB-231 cells, we found that the polyubiquitination of TβRI was decreased (Figure 3H). However, TβR1 degradation progressed when the cells were treated with the lysosome pathway inhibitor NH_4_Cl (Figure 3F). Together, these results suggest that depleting RAB1B potentiates TGF-β/SMAD signaling by inhibiting UPS-induced TβR1 degradation.

### Knockdown of RAB1B promotes TGF-β-induced epithelial-mesenchymal transition (EMT) characteristics in MCF10A cells

**Figure 4:**
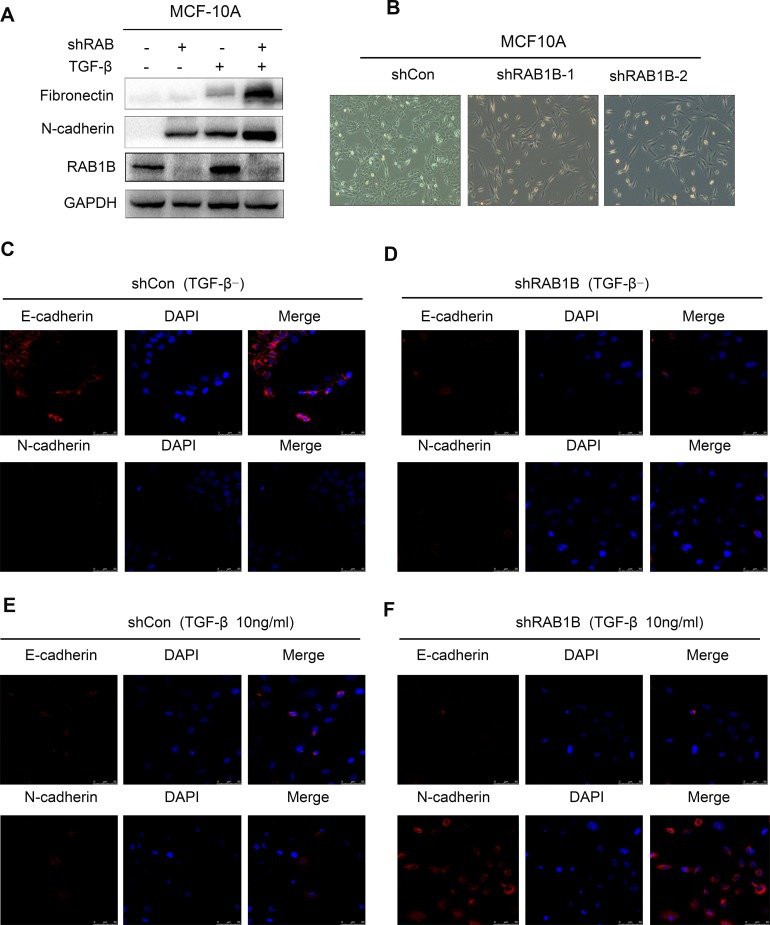
Knockdown of RAB1B promotes TGF-β-induced EMT **A.** MCF10A shCon and shRAB1B cell lysates were blotted with the indicated antibodies after treatment with or without TGF-β (10 ng/ml) for 48 h. **B.** Representative images showing the morphological change from an epithelial to a mesenchymal cell shape in MCF10A cells. **C.**, **D.**, **E.** and **F.** Representative images showing depleting RAB1B in MCF10A cells decreased levels of epithelial markers, E-cadherin and increased levels of mesenchymal markers, N-cadherin after treatment with or without TGF-β (10 ng/ml) for 48 h.

TGF-β/SMAD-induced EMT is a relatively well-established process during tumor progression [14]. Therefore, we assessed whether RAB1B knockdown induced the EMT program or enhanced TGF-β-induced EMT. RAB1B was knocked down in MCF10A cells, and the cells were left untreated or treated with TGF-β (10 ng/ml) for 48 h. In RAB1B stably knocked down MCF10A cells, a clear morphological change from an epithelial to a mesenchymal cell shape was observed (Figure 4A). Western blotting (Figure 4B) and Immunofluorescence (Figure 4C-4F) further showed that low expression of RAB1B potentiated TGF-β-induced changes in the expression of EMT markers, indicating that loss of RAB1B promotes EMT by cooperating with basal TGF-β signaling.

### Low RAB1B expression promotes breast cancer metastasis *in vivo*

**Figure 5:**
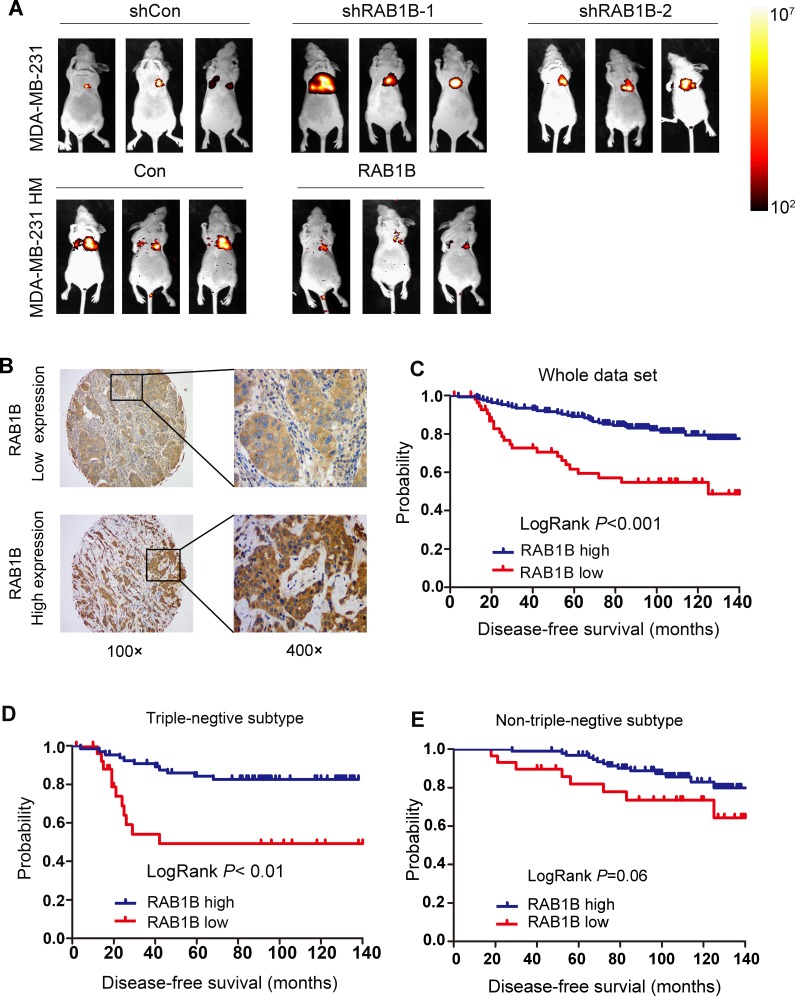
Low RAB1B expression promotes breast cancer metastasis *in vivo* and correlates with poor patient prognosis **A.** Representative BLI images of nude mice at the sixth week after tail vein injection of RAB1B downregulated MDA-MB-231 cells and RAB1B overexpressing MDA-MB-231HM cells.. **B.** Representative images of RAB1B immunohistochemistry are shown in the large (400 × magnification) and small images (100 × magnification). **C.** Kaplan-Meier curves showing that the disease-free survival of individuals with low RAB1B expression was significantly lower than that in individuals with high RAB1B expression (*P* < 0.001 by LogRank test). **D.** Cumulative DFS curves of patients with triple-negative breast cancer (*P* < 0.001 by LogRank test). **E.** Cumulative DFS curves of patients with non-triple-negative breast cancer (*P* = 0.06 by LogRank test).

Next, we labeled RAB1B knockdown MDA-MB-231 cells and RAB1B overexpressing MDA-MB-231HM cells with a retroviral construct expressing a GFP/luciferase fusion protein [15], and the *in vivo* metastasis capability of these cells was monitored by non-invasive bioluminescent imaging (BLI) six weeks after intravenous tail vein injection into nude mice. These data showed that RAB1B down-regulation significantly accelerated the development of lung metastases (Figure 5A). According to the BLI quantification, the metastasis burden caused by RAB1B knockdown cells was nearly 10-fold higher than that of control cells six weeks after injection. These findings confirm our hypothesis that low RAB1B expression promotes the metastasis of breast cancer cells *in vivo*.

### Low RAB1B expression correlates with poor patient prognosis

To evaluate the clinical importance of RAB1B in breast cancer, we performed an immunohistochemical analysis of TMAs containing samples from 250 breast cancer patients. Of these cases, 2 patients lacked follow-up data. Thus, the remaining 248 cases were included in the subsequent analysis. The clinicopathological characteristics of the cohort are summarized in Table 1. Because RAB1B expression varied across the breast tumor samples, we categorized RAB1B expression into low intensity and high intensity groups (Figure 5B). Kaplan-Meier survival curves showed that low RAB1B expression correlated with decreased survival in the whole data set and patients with triple-negative breast tumors (Figure 5C-5D), whereas, in patients with non-triple-negative breast tumors, RAB1B expression did not correlate with disease-free survival probability (Figure 5E), indicating that low RAB1B expression served as an independent predictor of poor disease-free survival in triple-negative breast cancer patients. Moreover, the univariate analysis indicated that low RAB1B expression at diagnosis was associated with a higher risk for disease relapse (hazard ratio (HR) = 0.323, 95% confidence interval (CI) 0.186-0.562; *P* < 0.001); further multivariate COX analysis exhibited a similar trend as the univariate analysis (Table 2).

**Table 1:** Correlation between tissue RAB1B expression in breast cancer patients and their clinicopathologic characteristics

Characteristics	Rab1b			
	Low	High	Total	*P^a^*
Age				0.470
≤50 years	31	81	112	
＞50 years	28	91	119	
NA			17	
Menopausal status				0.773
Premenopausal	27	75	102	
Postmenopausal	32	97	127	
NA			17	
Timor size				0.917
≤2 cm	26	78	104	
＞2 cm	32	93	125	
NA			19	
Lymph node status				0.246
Negative	32	109	141	
Positive	26	62	88	
NA			19	
Histological grade				0.253
1	3	3	6	
2	41	110	151	
3	12	46	58	
NA			33	
ER status				0.681
Negative	32	98	130	
Positive	27	73	100	
NA			18	
PR status				0.506
Negative	41	125	166	
Positive	18	44	62	
NA			20	
HER-2 status				0.050
Negative	42	97	139	
Positive	17	74	91	
NA			18	

Notes: ER: estrogen receptor; PR: progesterone receptor; HER-2: human epidermal growth factor receptor 2; *P^a^* was calculated using Pearson χ^2^ test. *P* < 0.05, which was considered to have a significant difference

**Table2:** Univariate and multivariate survival analysis of RAB1B expression in breast cancers

	Univariate analysis		Multivariate analysis	
	HR (95 % CI)	*P*-value	HR (95 % CI)	*P*-value
Age	0.978 (0.573-1.668)	0.935	0.598 (0.277-1.290)	0.190
Menopausal status	1.619 (0.926-2.833)	0.091	2.880 (1.232-6.729)	**0.015**
Timor size	1.373 (0.795-2.371)	0.256	1.630 (0.868-3.062)	0.129
Lymph node status	2.330 (1.356-4.005)	**0.002**	2.181 (1.212-3.925)	**0.009**
Histological grade	1.715 (1.013-2.906)	**0.045**	1.879 (1.031-3.427)	**0.039**
ER status	0.815 (0.471-1.409)	0.464	1.380 (0.614-3.099)	0.435
PR status	0.484 (0.228-1.026)	0.058	0.340 (0.131-0.884)	**0.027**
HER-2 status	0.915 (0.530-1.582)	0.752	1.126 (0.579-2.190)	0.727
Rab1b	0.323 (0.186-0.562)	**0.000**	0.244 (0.132-0.452)	**0.000**

Notes: ER: estrogen receptor; PR: progesterone receptor; HER-2: human epidermal growth factor receptor 2; CI: confidence interval; HR: hazard ratio; Bold values are statistically significant

## DISCUSSION

In this report, we demonstrate that the RAB1B-TβR1 interaction represents a critical mechanism for controlling TGF-β signaling and breast cancer cell invasion and metastasis. Tumor cells typically secrete abundant amounts of TGF-β, which promotes invasion and metastasis [16]. Indeed, some studies have suggested that anti-TGF-β therapies are efficacious in inhibiting cancer invasion and metastasis in animal models [17]. Moreover, some clinical agents that target this pathway have shown promising results [18]. Although the TGF-β signaling pathway is considered a promising therapeutic target, its multifunctional action in various biological processes makes it a very challenging target [19]. Because most of the current TGF-β signaling inhibitors are developed for suppressing all TGF-β responses, a wide range of side effects may occur. However, our data demonstrate that RAB1B can regulate TβR1 degradation, which may allow for selective targeting of the TGF-β signaling pathway. This finding led to the identification of RAB1B as a potential biomarker for TGF-β-dependent metastasis in breast cancer.

Our results also demonstrate that the role of RAB1B in inhibiting the degradation of TβR1 serves as an underlying mechanism by which RAB1B regulates TGF-β-SMAD3-induced EMT. Depleting RAB1B in breast cancer cells inhibited the action of TβR1 protein ubiquitination and thereby potentiated TGF-β signaling. Nevertheless, the mechanisms contributing to the correlation between RAB1B and TβR1 degradation require further investigation to determine whether this correlation is related to RAB1B-mediated vesicle trafficking. RAB1B is a member of the RAB family of small GTPases with a well-characterized function in regulating intercellular vesicle trafficking in endocytic pathways [20]. Endocytosis entails the selective packaging of cell surface proteins, such as receptors for cytokines and adhesion components, in cytoplasmic vesicles (endosomes). A series of sorting events that determine the fate of proteins, either degradation or recycling back to the plasma membrane, rely on intrinsic sequence motifs, posttranslational modifications (e.g., ubiquitination) and transient assemblies of both RAB GTPases and phosphoinositide-binding proteins [21, 22]. Chung-Han Tsai et al also reported that RAB37 acted as metastasis suppressor by promoting TIMP1 secretion, indicating that RAB-controlled trafficking pathways were altered during tumorigenesis [23]. Indeed, RAB1A, which is highly homologous to RAB1B, was reported to regulate the recycling and localization of integrin β1 and thereby control cell migration ability [24]. As we observed an interaction between RAB1B and TβR1, this mechanism should be further examined in future studies.

Previous results have demonstrated that SMAD3 and SMAD4 are critical for TGF-β-induced EMT and the metastasis of breast cancer cells [25]. Accordingly, our data show that upon RAB1B depletion in breast cancer cells, the TβR1-SMAD3 signaling pathway was activated, leading to EMT induction. EMT is thought to drive invasion and metastasis in breast cancer and other epithelial cancers and potentiate the generation of cells with stem cell-like characteristics [26]. Our results also indicate that a loss of RAB1B in MCF10A cells led to a morphology change consistent with EMT and the up-regulation of fibronectin and N-cadherin, which are well-characterized markers of early EMT induction. Thus, the loss of RAB1B may play an early role in the initiation of EMT and could serve as an important biomarker of aggressive disease.

In addition, we observed that some breast cancer samples expressed aberrantly low levels of RAB1B and that the loss of RAB1B expression in patient tissue arrays was correlated with a poor prognosis in breast cancer patients. Furthermore, in mouse models, we demonstrated that RAB1B suppressed the metastatic potential of breast cancer cells. Importantly, RAB1B gene copy number variation is also found in many other types of cancer, such as human hepatocellular carcinomas and colon cancers, suggesting that the oncogenic role of RAB1B is not limited to breast cancer. Indeed, RAB1B was shown to be up-regulated in 11 hepatocellular carcinoma cases and 1 cholangiohepatoma case [27]. High frequencies of copy number variation of RAB1B (9/98) were also reported in 98 human hepatocellular carcinoma tissues [28], and RAB1B is known to suppress colon tumor growth both *in vitro* and *in vivo* by targeting miR-502 [29]. Taken together, these data indicate the potential importance of RAB1B for the initiation or progression of different tumor types, although these roles need to be further clarified in future studies.

In conclusion, using a combination of *in vivo* and *in vitro* functional metastasis assays and extensive clinical correlation analysis, we identify RAB1B as a novel tumor suppressor involved in human breast cancer. The results presented here provide a paradigm for the RAB1B-mediated regulation of both TβR1 protein levels and TGF-β-induced EMT. In the presence of RAB1B, TβR1 is progressively degraded and the oncogenic, EMT-promoting functions of TGF-β are restrained. However, loss of RAB1B results in elevated TβR1, which is then subjected to SMAD3 phosphorylation and nuclear translocation induced by TGF-β to activate EMT. In recent years, many studies have demonstrated various TGF-β/Smad pathway antagonists are being partial successfully used in clinical trials [30, 31]. As our study showed RAB1B can inhibit TGF-β/Smad pathway, RAB1B may combine with other TGF-β/Smad pathway antagonists to treat metastastic TNBC. Our findings contribute novel data showing that RAB1B may serve as a novel and important biomarker to stratify patients with advanced breast cancer for more effective treatments with specific targeted therapies aimed at the TGF-β-SMAD3 pathway.

## MATERIALS AND METHODS

### Study population

This study involved 250 breast cancer patients with a diagnosis of pathologically invasive ductal breast cancer and a follow-up period of at least 5 years. The diagnoses were verified by two independent pathologists in the Department of Pathology of Fudan University Shanghai Cancer Center (FDUSCC, Shanghai, China). The specimens from these patients were collected by the Department of Breast Surgery of FDUSCC from August 2001 to March 2006. This study was approved by the Ethics Committee of FDUSCC, and each participant signed an informed consent document.

### Tissue microarrays (TMAs)

TMAs were constructed from archival formalin-fixed, paraffin wax-embedded samples of carcinomas obtained from the 250 breast cancer patients described above. Tissue cylinders, with a diameter of 2 mm, were punched from the previously marked tumor area of each block (donor block) and inserted into a recipient paraffin wax block, resulting in a 10×10 array. Tissue samples from all 250 patients were punched twice into the microarray to compare the staining patterns in different areas of the same tumor.

### iTRAQ-nano-HPLC-MS/MS analyses

The cell lysates of parental MDA-MB-231 breast cancer cells and highly metastatic MDA-MB-231 cells (MDA-MB-231HM) were quantified using a Bradford assay, labeled with iTRAQ labeling reagents (Applied Biosystems), and digested with trypsin. The peptides were fractionated on a Waters ultra performance liquid chromatography (UPLC) device, and the fraction was then separated by nanoscale high-performance liquid chromatography (nano-HPLC) (Eksigent Technologies) on a secondary reverse-phase (RP) analytical column. A Triple TOF 4600 mass spectrometer (MS) was operated in information-dependent data acquisition mode to switch automatically between MS and tandem MS (MS/MS) acquisition. MS/MS spectra were extracted and charge state deconvoluted using an MS Data Converter from AB Sciex.

### Cell culture

All of the breast cancer cell lines, normal breast MCF10A cells and HEK 293T cells were obtained from the American Type Culture Collection (Manassas, VA, USA) and maintained under conditions specified by the provider. All of the cells were cultured in a 5% CO_2_ incubator at 37°C.

### Western blot analysis

Whole-cell lysates were generated using Pierce T-PER (Tissue Protein Extraction Reagent; Thermo Fisher Scientific Inc.) containing protease inhibitor cocktail tablets (Roche) and phosphatase inhibitors (Roche). In total, 30 μg of the cell lysates were resolved by sodium dodecyl sulfate polyacrylamide gel electrophoresis (SDS-PAGE) and transferred to polyvinylidene fluoride (PVDF) membranes (Pall). The membranes were blocked in 5% milk or 5% bovine serum albumin and then incubated with various primary antibodies followed by the appropriate horseradish peroxidase (HRP)-conjugated secondary antibodies. Immunoreactive bands were identified using enhanced chemiluminescence, according to the manufacturer's instructions, and quantified by densitometry. The antibodies used in this study are listed in Supplementary Table S1.

### Quantitative real-time PCR

Total RNA was extracted with TRIzol reagent (Invitrogen Corporation) and reverse transcribed using the PrimeScript RT Reagent Kit (Perfect Real Time; TaKaRa Biotechnology). Subsequently, real-time PCR was performed with SYBR Premix Ex Taq (TaKaRa Biotechnology) using an ABI Prism 7900 instrument (Applied Biosystems). The primer sequences used in this study are as follows:

GAPDH: F 5′-GGTGGTCTCCTCTGACTTCAACA-3′,
GAPDH: R 5′-GTTGCTGTAGCCAAATTCGTTGT-3′,
RAB1B: F 5′-AGATCCGAACCATCGAGCTG-3′,
RAB1B: R 5′-GCGTAGGATTCCTGG TCAGTG-3′,
TβR1: F 5′-CCCTGGACACCAACTATTGC-3′,
TβR1: R 5′- CTTCCAGCCGAGGTCCTT-3′,
SMAD3: F 5′-AGACCCCACCCCCTGGCTACCTG-3′,
SMAD3: R 5′-GGGGACATCGGATTCGGGGA-3′,
SMAD7: F 5′-TGGATGGCGTGTGGGTTTA-3′,
SMAD7: R 5′-TGGCGGACTTGATGAAGATG-3′.

### Plasmids and short hairpin RNA (shRNA)

Human RAB1B cDNA was subcloned from the breast cancer cell line MDA-MB-231 into the lentiviral vector pCDH-CMV-MCS-EF1-Puro with a Flag tag. The cloned primer sequence is as follows:

F:5′-CCGgaattcGCCACCATGGACTACAAGGACGATGATGACAAGCTCGATG-GAGGAATGAACCCCGAATATGACTA-3′;
R: 5′-CGCggatccCTAGCAACAGCCACCGCCAG-3′.

RAB1B shRNAs and the negative control were purchased from GeneChem and expressed in the GV248 backbone. The target sequences are as follows:

shRNA-1: 5′-TCATCGTGGTGTATGACGT-3′.
shRNA-2: 5′-CCATCACTTCCAGCTACTA-3′.

### Lentivirus packaging and infection

Briefly, 293T cells were co-transfected with lentiviral vectors and the packaging vectors PCDH (or GV248), psPAX2 and pMD2G. Forty-eight hours after transfection, the viral supernatants were collected, filtered and concentrated by ultracentrifugation. Polybrene (Sigma-Aldrich, Natick, MA, USA) was added at a working concentration of 8 μg/ml. The cells were incubated with virus for 12 h, and then media containing fetal bovine serum (FBS) was applied. Twenty-four hours later, the infected cells were subjected to selection with 2 μg/ml puromycin for one week.

### Transwell assays

Cells (5×10^5^ for the migration assay and 10×10^5^ for the invasion assay) were plated in the top chamber of a non-coated membrane or Matrigel-coated transwell chambers (BD Biosciences) in media without FBS. Media supplemented with serum was used as a chemoattractant in the lower chamber. The cells were incubated for 15-20 h, and the cells that did not migrate through the pores were removed with a cotton swab. The cells on the lower surface of the membrane were stained with methanol and 0.1% crystal violet and then counted.

### Kinetic wound-healing assay

Breast cancer cells (3.5×10^4^) were plated on 96-well plates (Essen ImageLock, Essen Instruments), and a wound was scratched with wound scratcher (Essen Instruments). Wound confluence was monitored with Live-Cell Imaging System and software (Essen Instruments). Wound closure was observed every 2 hours for 28 hours by comparing the mean relative wound density of at least three biological replicates in each experiment.

### Immunofluorescence

MCF10A stable cell lines grown on coverslips were fixed with 4% paraformaldehyde for 30 min at room temperature, permeabilized with 0.5% Triton X-100 for 5 min at 4°C, and incubated with primary antibodies for 2 h at 37°C. The slides were then incubated with Alexa 695-conjugated (red, Abcam) or Alexa 594-conjugated (red, Invitrogen) secondary antibodies for 40 minutes at room temperature. Images were captured with a confocal laser microscope (Leica TCS SP5 II). At least 100 cells were analyzed for each group.

### Phosphoprotein profiling with the Phospho Explorer antibody microarray

The Phospho Explorer antibody microarray, which was designed and manufactured by Full Moon Biosystems, Inc. (Sunnyvale, CA), contains 1,318 antibodies. Each of these antibodies includes two replicates that are printed on a coated glass microscope slide, along with multiple positive and negative controls. The antibody array experiment was performed according to an established protocol.

### Metastasis assays in nude mice

All animal work was performed in accordance with the guidelines of the Institutional Animal Care and Use Committee of Fudan University under approved protocols. In total, 4×10^5^ cells were washed in phosphate-buffered saline (PBS) and injected intravenously into female BALB/c nude mice (*n* = 6) to study lung metastasis activity. Noninvasive bioluminescence imaging was performed to quantify the metastasis burden in the target organs using an *in vivo* imaging system (Bruker MI).

### Statistical analysis

The results were reported as the mean ± SD or mean ± SEM, as indicated in the figure legends. The results were analyzed using SPSS 16.0 software (SPSS, Chicago, IL, USA) and PRISM 5.0 (GraphPad Software Inc., San Diego, CA, USA). Comparisons of quantitative data between two groups were analyzed with Student's *t*-test (two-tailed; *P* < 0.05 was considered significant). The χ^2^ test was used to compare qualitative variables. The cumulative survival time was calculated using the Kaplan-Meier method and analyzed using the log-rank test. Univariate and multivariate analyses were based on the Cox proportional hazards regression model. *P* values < 0.05 were considered statistically significant.

## SUPPLEMENTARY MATERIAL TABLE AND FIGURE


