# Supplementary material for: Loss of RAB1B promotes triple-negative breast cancer metastasis by activating TGF-β/SMAD signaling
Source: Oncotarget. 2015 Apr 19;6(18):16352–65. doi: 10.18632/oncotarget.3877 (PMC4599274; doi:10.18632/oncotarget.3877)
Supplement: Supplementary file 1 [file oncotarget-06-16352-s001.pdf]

Loss of RAB1B promotes triple-negative breast cancer metastasis by activating TGF-β/SMAD signaling

Supplementary Material

Supplementary Table 1: Antibodies used in this study

Primary Antibody	Clone, source	Dilution	Company
RAB1B	Rabbit monoclonal	1:1000 for WB	Proteintech
p-Smad3 (S423 + S425)	Rabbit monoclonal	1:1000 for WB	Abcam
SMAD3	Rabbit monoclonal	1:1000 for WB	Abcam
TβR1	Rabbit polyclonal	1:100 for WB	Santa Cruz (Santa Cruz, CA)
N-cadherin	Mouse monoclonal	1:1000 for WB	BD Biosciences
Fibronectin	Rabbit polyclonal	1:1000 for WB	Proteintech
GAPDH		1:30000 for WB	Proteintech
**Second Antibody**	**Clone, source**	**Dilution**	**Company**
Secondary antibody	HRP conjugated goat anti mouse IgG	1:3000 for WB	Proteintech
Secondary antibody	HRP conjugated goat anti rabbit IgG	1:3000 for WB	Proteintech

WB: Western blotting

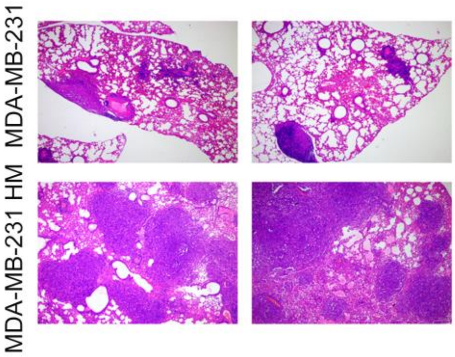

Supplementary Figure S1: Representative HE staining images of nude mice lungs at the sixth week after tail vein injection of MDA-MB-231 and MDA-MB-231HM cells.
